# Supplementary material for: Contextual and psychosocial factors influencing caregiver safe disposal of child feces and child latrine training in rural Odisha, India
Source: PLoS One. 2022 Sep 9;17(9):e0274069. doi: 10.1371/journal.pone.0274069 (PMC9462565; doi:10.1371/journal.pone.0274069)
Supplement: S1 Table — +The sample size varies by demographic variable, primarily due to respondents ending the survey early. One respondent said “don’t know” for age. Here are the number of surveys with missing data by variable: 9 for drinking water, enclosed bathing area, two household latrines, and share latrine; 10 for latrine in use; 11 for education, religion, employment, and caste; 12 for household size; 14 for caregiver latrine use; 20 for latrine has piped water and location; 36 for latrine structure; and 52 missing for number of pits. (DOCX) [file pone.0274069.s001.docx]

**S1 Table. Caregiver and household demographics (N = 791).**

| **Participant characteristics** | **N^+^** | **%** |
| --- | --- | --- |
| **Mother of child** | 785 | 99% |
| **Mean age in years** (SD) | 26.78 | 5.16 |
| **Education** |  |  |
| Never attended school | 157 | 20% |
| Primary/upper primary (grades 1-8) | 268 | 34% |
| Secondary/senior secondary (grades 9-12) | 312 | 40% |
| Graduate/post-graduate (13+) | 37 | 5% |
| Don’t know/refused | 6 | 1% |
| **Employment Status** |  |  |
| Unemployed | 448 | 57% |
| Self-employed (i.e. subsistence agriculture) | 273 | 35% |
| Employed (i.e. work outside home) | 59 | 8% |
| **Last defecation used latrine** | 558 | 72% |
| **Household characteristics** | **N** | **%** |
| **Mean household size** (SD) | 5.87 | 2.2 |
| **Mean number of household members that help with childcare** (SD) | 2.69 | 1.79 |
| **Multiple children <5 years** | 147 | 19% |
| **Household religion** |  |  |
| Hindu | 645 | 83% |
| Christian | 129 | 17% |
| No religion | 2 | <1% |
| Other | 2 | <1% |
| Refused | 2 | <1% |
| **Household caste** |  |  |
| Other backward caste (OBC) | 288 | 37% |
| Scheduled tribe | 183 | 23% |
| General | 144 | 18% |
| Scheduled caste | 65 | 8% |
| Other | 72 | 9% |
| Don't know/refused | 28 | 4% |
| **WASH characteristics** | **N** | **%** |
| **Functional piped water to household** | 701 | 90% |
| **Mean hours without piped water** (SD) | 9.97 | 10.43 |
| **Enclosed bathing area** | 763 | 98% |
| **Latrine characteristics** |  |  |
| Functional piped water inside | 391 | 51% |
| Structure fully intact | 617 | 82% |
| Two pits | 499 | 68% |
| In/near household (<50ft) | 676 | 88% |
| **Two household latrines (vs. one)** | 33 | 4% |
| **Share latrine with other households** | 43 | 6% |

+The sample size varies by demographic variable, primarily due to respondents ending the survey early. One respondent said “don’t know” for age. Here are the number of missing data by variable: 9 for drinking water, enclosed bathing area, two household latrines, and share latrine; 11 for education, religion, employment, and caste; 12 for household size and childcare; 14 for caregiver latrine use; 20 for location; 30 for mean hours without water and latrine piped water; 36 for latrine structure; and 52 missing for number of pits.
