# Supplementary material for: Contextual and psychosocial factors influencing caregiver safe disposal of child feces and child latrine training in rural Odisha, India
Source: PLoS One. 2022 Sep 9;17(9):e0274069. doi: 10.1371/journal.pone.0274069 (PMC9462565; doi:10.1371/journal.pone.0274069)
Supplement: S2 Table — (DOCX) [file pone.0274069.s002.docx]

**S2 Table. Descriptive statistics and bivariate regressions between caregiver safe disposal intention and predictor variables.**

| **Variable** | **N** | **Mean/n** | **SD/%** | **b** | **p-value** |
| --- | --- | --- | --- | --- | --- |
| Safe disposal intention | 299 | 3.92 | 1.41 | - | - |
| *Caregiver characteristics* |  |  |  |  |  |
| Caregiver's age (years) | 298 | 25.78 | 5.23 | 0.006 | 0.739 |
| Caregiver's years of education | 292 | 6.55 | 4.18 | 0.035 | 0.056 |
| Caregiver unemployed | 298 | 183 | 61% | 0.106 | 0.526 |
| Caregiver latrine use | 295 | 174 | 59% | 0.212 | 0.204 |
| *Household characteristics* |  |  |  |  |  |
| Christian household | 298 | 50 | 17% | 0.113 | 0.606 |
| General Caste | 298 | 50 | 17% | ref | ref |
| Schedule Caste | 298 | 26 | 9% | 0.529 | 0.122 |
| Other Backward Caste | 298 | 103 | 35% | 0.182 | 0.454 |
| Scheduled Tribe | 298 | 70 | 23% | 0.260 | 0.320 |
| Other/Don't know caste | 298 | 49 | 16% | 0.097 | 0.733 |
| **Household wealth quintile** | 297 | 3.07 | 1.41 | 0.139 | **0.017** |
| Single child <5 in household | 299 | 256 | 86% | 0.015 | 0.949 |
| Childcare support size | 297 | 2.81 | 1.74 | 0.010 | 0.839 |
| *WASH characteristics* |  |  |  |  |  |
| Hours without piped water | 287 | 9.48 | 10.40 | -0.014 | 0.085 |
| **Latrine in/near household (<50ft)** | 294 | 263 | 89% | -0.661 | **0.013** |
| Latrine has functional piped water | 292 | 156 | 53% | 0.008 | 0.959 |
| Latrine structure fully intact | 290 | 228 | 79% | 0.195 | 0.334 |
| Two pits | 282 | 186 | 66% | 0.013 | 0.943 |
| *Child characteristics* |  |  |  |  |  |
| **Child's age (months)** | 299 | 15.09 | 10.00 | -0.023 | **0.005** |
| Female child | 299 | 133 | 44% | -0.208 | 0.206 |
| **Child is ambulatory** | 299 | 161 | 54% | -0.405 | **0.013** |
| *Social support factors* |  |  |  |  |  |
| Emotional support | 289 | 3.30 | 1.45 | 0.067 | 0.238 |
| **Instrumental support** | 297 | 4.62 | 1.32 | 0.123 | **0.045** |
| **Informational support** | 291 | 3.40 | 1.81 | 0.121 | **0.008** |
| *RANAS psychosocial factors* |  |  |  |  |  |
| Health knowledge | 299 | 181 | 61% | 0.203 | 0.223 |
| Diarrhea health knowledge | 299 | 134 | 45% | 0.064 | 0.695 |
| **Perceived vulnerability (1)** | 293 | 3.34 | 1.51 | 0.120 | **0.027** |
| Perceived vulnerability (2) | 295 | 2.40 | 1.52 | 0.010 | 0.848 |
| Perceived severity | 294 | 3.52 | 1.47 | 0.109 | 0.050 |
| **Positive attitudes (safe disposal)** | 291 | 3.65 | 1.06 | 0.695 | **<0.001** |
| **Negative attitudes (unsafe disposal)** | 294 | 2.78 | 1.22 | 0.328 | **<0.001** |
| Benefits of child OD | 294 | 4.16 | 1.37 | 0.073 | 0.224 |
| Disgust latrine | 290 | 4.35 | 1.25 | 0.019 | 0.771 |
| **Personal norm** | 297 | 3.85 | 1.21 | 0.429 | **<0.001** |
| **Village descriptive norm** | 290 | 2.19 | 1.78 | 0.194 | **<0.001** |
| **Personal norm (motherhood)** | 297 | 4.61 | 0.97 | 0.333 | **<0.001** |
| **Household injunctive norm** | 296 | 3.97 | 1.55 | 0.465 | **<0.001** |
| General injunctive norm (approval) | 295 | 2.57 | 1.25 | 0.032 | 0.626 |
| **Village injunctive norm** | 299 | 201 | 67% | 0.564 | **0.001** |
| **Self-efficacy** | 297 | 3.41 | 1.16 | 0.539 | **<0.001** |
| Action control | 298 | 3.42 | 1.69 | -0.061 | 0.210 |
| **Barrier planning** | 298 | 188 | 63% | 1.131 | **<0.001** |
| **Commitment** | 294 | 3.61 | 1.44 | 0.650 | **<0.001** |
