# Supplementary material for: Contextual and psychosocial factors influencing caregiver safe disposal of child feces and child latrine training in rural Odisha, India
Source: PLoS One. 2022 Sep 9;17(9):e0274069. doi: 10.1371/journal.pone.0274069 (PMC9462565; doi:10.1371/journal.pone.0274069)
Supplement: S3 Table — (DOCX) [file pone.0274069.s003.docx]

**S3 Table.** **Descriptive statistics and bivariate regressions between latrine training intensity and predictor variables.**

| **Variable** | **N** | **Mean/n** | **SD/%** | **b** | **p-value** |
| --- | --- | --- | --- | --- | --- |
| Latrine training intensity | 503 | 3.39 | 1.85 | - | - |
| *Caregiver characteristics* |  |  |  |  |  |
| **Caregiver's age (years)** | 503 | 27.36 | 5.02 | -0.047 | **0.004** |
| **Caregiver's years of education** | 492 | 6.60 | 4.19 | 0.082 | **<0.001** |
| **Caregiver unemployed** | 493 | 270 | 55% | 0.349 | **0.036** |
| **Caregiver latrine use** | 493 | 389 | 79% | 1.747 | **<.001** |
| *Household characteristics* |  |  |  |  |  |
| Christian household | 491 | 81 | 16% | 0.360 | 0.110 |
| General Caste | 492 | 97 | 20% | ref |  |
| Schedule Caste | 492 | 114 | 23% | -0.022 | 0.950 |
| Other Backward Caste | 492 | 190 | 39% | 0.128 | 0.580 |
| Scheduled Tribe | 492 | 39 | 8% | 0.012 | 0.962 |
| Other/Don't know caste | 492 | 52 | 11% | 0.209 | 0.513 |
| **Household wealth quintile** | 493 | 3.13 | 1.39 | 0.193 | **0.001** |
| Single child <5 in household | 503 | 388 | 77% | 0.085 | 0.664 |
| Childcare support size | 493 | 2.62 | 1.82 | 0.001 | 0.988 |
| *WASH characteristics* |  |  |  |  |  |
| **Hours without piped water** | 484 | 10.38 | 10.48 | -0.018 | **0.028** |
| **Latrine in/near household (<50ft)** | 488 | 421 | 86% | 0.543 | **0.025** |
| Latrine has functional piped water | 479 | 239 | 50% | 0.185 | 0.268 |
| **Latrine structure fully intact** | 476 | 393 | 83% | 0.820 | **<0.001** |
| Two pits | 468 | 319 | 68% | 0.150 | 0.415 |
| *Child characteristics* |  |  |  |  |  |
| Child's age (months) | 503 | 38.87 | 11.49 | 0.013 | 0.069 |
| Female child | 503 | 255 | 51% | 0.152 | 0.357 |
| Child able to squat on their own | 503 | 439 | 87% | 0.140 | 0.573 |
| Child speaks in full sentences | 503 | 398 | 79% | 0.370 | 0.069 |
| Child follows directions | 503 | 439 | 87% | 0.462 | 0.062 |
| *Social support factors* |  |  |  |  |  |
| **Emotional support** | 464 | 3.62 | 1.51 | 0.160 | **0.005** |
| **Instrumental support** | 478 | 4.51 | 1.45 | 0.329 | **<0.001** |
| **Informational support** | 467 | 3.52 | 1.76 | 0.130 | **0.007** |
| *RANAS psychosocial factors* |  |  |  |  |  |
| **Perceived vulnerability of child OD** | 482 | 3.55 | 1.59 | 0.293 | **<0.001** |
| Perceived vulnerability latrine use | 483 | 4.49 | 0.87 | -0.023 | 0.807 |
| Benefit- latrine training | 477 | 4.08 | 1.09 | 0.144 | 0.062 |
| **Unbeneficial for child OD** | 480 | 4.52 | 1.11 | 0.242 | **0.001** |
| **Safety concern w/ child OD** | 482 | 4.14 | 1.18 | 0.174 | **0.015** |
| Safety concern w/ latrine use | 483 | 3.86 | 1.48 | 0.047 | 0.408 |
| Time-consuming - latrine training | 481 | 3.32 | 1.24 | 0.132 | 0.051 |
| **Difficult - latrine training** | 482 | 4.24 | 1.24 | 0.337 | **<0.001** |
| **Like - latrine training** | 482 | 4.01 | 1.06 | 0.445 | **<0.001** |
| **Irritated - latrine training** | 481 | 4.05 | 1.24 | 0.223 | **0.001** |
| **Proud - latrine training** | 477 | 3.29 | 1.49 | 0.228 | **<0.001** |
| Helpless - latrine training | 473 | 4.16 | 1.34 | 0.096 | 0.130 |
| **Personal norm** | 481 | 4.06 | 1.08 | 0.299 | **<0.001** |
| **Personal norm (age to train)** | 483 | 337 | 70% | 0.493 | **0.007** |
| **Personal norm (father's role)** | 482 | 3.71 | 1.15 | 0.324 | **<0.001** |
| **Village descriptive norm** | 477 | 2.91 | 1.74 | 0.221 | **<0.001** |
| **Household injunctive norm** | 483 | 4.54 | 1.07 | 0.383 | **<0.001** |
| Belief about role of fathers | 481 | 4.59 | 1.07 | 0.146 | 0.062 |
| **Self-efficacy** | 479 | 4.00 | 0.87 | 0.601 | **<0.001** |
| **Action control** | 481 | 3.92 | 1.41 | 0.808 | **<0.001** |
| **Commitment** | 470 | 3.80 | 1.26 | 0.283 | **<0.001** |
| **Intention** | 479 | 4.51 | 0.91 | 0.594 | **<0.001** |
